# Improved electrode stimulation stability of Utah arrays^*^

**DOI:** 10.1186/s42234-025-00190-9

**Published:** 2025-12-27

**Authors:** Taylor Stump, Brian Baker, Ryan Caldwell, Rohit Sharma, Sandeep Negi, Loren Rieth

**Affiliations:** 1 Department of Chemical and Biomedical Engineering, West Virginia University, Morgantown, WV 26506 USA; 2 Utah Nanofabrication Lab, University of Utah, Salt Lake City, UT 84112 USA; 3 Department of Biomedical Engineering, University of Utah, Salt Lake City, UT 84112 USA; 4 Avanos Medical, Alparetta, GA 30004 USA; 5 Applied Biosensors, Salt Lake City, UT 84115 USA; 6 Blackrock Neurotech, Salt Lake City, UT 84108 USA; 7 Electrical and Computer Engineering Department, University of Utah, Salt Lake City, UT 84112 USA; 8 Mechanical, Materials and Aerospace Engineering Department, West Virginia University, Morgantown, WV 26506 USA

**Keywords:** Stimulation, Microelectrode, Lifetime, Metallization, Brain-computer interface, Iridium oxide, Stability

## Abstract

**Background:**

The Utah electrode array (UEA) is a promising microelectrode technology with potential applications to assist patients with sensory loss, spinal cord injuries, and limb loss serving as neural prosthetics and brain-computer interfaces. Performance lifetime of microelectrodes, particularly when used for stimulation, remains one of the challenges for their clinical translation.

**Methods:**

This study characterizes the stimulation stability of an optimized iridium oxide (IrOx) *research* metallization for the UEA in comparison to the Blackrock standard practice metallization. The stimulation stability (Stim-Stab) protocol used electrochemical characterization and either 10^6^ or 4 × 10^6^ pulses at 2,100 *µ*A (420 nC/ph) with longitudinal voltage transient measurements and physical characterization to quantify electrode lifetime.

**Results:**

Approximately 50% of electrodes using the Blackrock standard practice process electrical failed during 10^6^ pulses, whereas no electrical failures were observed from 4 × 10^6^ pulses for the *research* metallization. Backscattered scanning electron microscopy (BSEM) determined that all electrodes with electrical failures during Stim-Stab testing had complete loss of metallization. Of electrodes with good electrical outcomes from Stim-Stab testing 80% and 50% of those had notable metallization damage for *BRM* and *research* metallization, respectively.

**Conclusions:**

These results suggest that the *research* metallization makes important steps for improving electrode metallization lifetime of Utah arrays. Additionally, the Stim-Stab method provides valuable feedback for engineering improved electrode metallization.

**Supplementary Information:**

The online version contains supplementary material available at 10.1186/s42234-025-00190-9.

## Background

Microelectrode technologies such as the Utah electrode array (UEA) have tremendous potential to partially restore sensory and motor function after spinal cord injuries (Russman et al. 2025; Flesher et al. 2016), sensory loss (e.g. hearing loss) (Middlebrooks and Snyder 2008; Badi et al. 2003; Thomas et al.^2024^), and improve performance in activities of daily living after amputations (Page et al. 2018; Wendelken et al. 2017; George et al. 2020) by interfacing with the central and peripheral nervous systems to record and stimulate neural signals. Stimulation with microelectrodes remains a significant challenge due to the increased current densities required for smaller electrode surface areas (Cogan et al. 2016; Cogan 2008). Thus, despite the ability to evoke sensory percepts at currents on the scale of tens of *µ*A (Page et al. 2021; George et al. 2019), the current densities compared to macro-scale cuff and disc electrodes (e.g. ECoG grids) can be large. This motivates research to improve the charge injection capacity, impedance, and stimulation stability of electrode materials, and the associated encapsulation materials that define the electrode area (Caldwell et al. 2020). The amount of charge an electrode can deliver without degradation is a critical aspect of the stimulation stability for such microelectrodes. Degradation processes that occur in quiescent and stimulated electrodes after long-term in-dwelling placement in human subjects was recently analyzed (Bjånes et al. 2025). The study found degradation in some electrodes with Pt or IrOx electrodes, but that degradation was not accelerated by stimulation at the levels used. Our role on the DARPA HAPTIX program was to maximize the stimulation lifetime of the IrOx tip metallization used for Utah Slanted electrode arrays (USEAs) such that reliable long-term (> 1 year) stimulation in peripheral nerves of human subjects (George et al. 2020) could be achieved. These studies delivered up to 19 mC of charge for some electrodes during nearly 1.5 years in-dwelling. This is a significant increase compared to previous levels of stimulation for clinical investigations with Utah arrays in peripheral nerves, but still far below the charge delivered by clinical devices (e.g. cochlear implants). This report uses accelerated aging method to evaluate the stimulation stability of electrodes similar to the one used to characterize polyimide electrodes (Sun et al. 2023). This method is used to quantify the increased stimulation lifetime resulting from improvement to the IrOx tip metal stack.

This study involves quantitative comparison of UEAs that use the current practice (*ca.* 2019) Blackrock metallization compared to the *research* metallization process developed by this team. A set of four 4 × 4 UEAs, two using the Blackrock standard practice metallization (*BRM*) and two using the *research* metallization, were fabricated and evaluated using a heavily accelerated stimulation stability protocol. This Stim-Stab protocol uses stimulation levels 21 × the maximum safe levels of 100 *μ*A (20 nC/phase) used physiologically for UEAs. The 100 µA intensity for chronic stimulation was found to not result in an increase tissue response in NHP models (Chen et al. 2014; Rajan et al. 2015; Kim et al. 2015). These pre-clinical studies were the basis of clinical investigations at University of Pittsburgh (Flesher et al. 2016), CalTech (Salas et al. 2018), and Applied Physics Laboratory (Fifer et al. 2022) with the Utah array, which also used these intracortical microstimulation limits. The 2100 *μ*A used in this accelerated aging study, the upper limit of our stimulator and measurement equipment, was chosen in order to provide a quick, practical, and quantitative worst-case method to evaluate improvements in electrode metallization by significantly accelerating the degradation mechanisms. This heavy acceleration significantly exceeds the water window and might result in activation of failure modes that would not occur under physiological use conditions but remain a useful comparison due to the significant differences in stability revealed by the lifetime analysis. The UEAs were evaluated using microscopy before and after stimulation to ensure the electrode surface areas were comparable and to characterize damage to the tip metallization. The electrochemical impedance spectra (EIS), cyclic voltammetry (CV), and voltage transients (VT) were also characterized before and after stimulation to determine the impact of the stimulation on these properties, and the combination of Stim-Stab, EIS, and electron microscopy outcomes was used to evaluate electrode lifetime. The *research* metallization was found to have a significantly improved stimulation lifetime.

## Methods

The following methods are part of the Stimulation-Stability (Stim-Stab) protocol used to evaluate the lifetime of neural electrodes under stimulation conditions.

### Utah electrode arrays

Four 4 × 4 UEAs where fabricated using methods that have been extensively reported in the literature (Negi et al. 2010a; Negi et al. 2010b; Jones et al. 1992; Campbell et al. 1991). Two of the arrays were fabricated using the Blackrock standard practice IrOx metallization, and two were fabricated with the *research* metallization being evaluated. The Blackrock standard process utilizes a co-sputtered 50 nm PtSi film as the primary layer to form the electrical contact with the Si shank, while the *research* metallization utilizes a pure 25 nm Pt layer. Both stacks then utilize similar sputtered deposited layers of Ir and IrOx to serve as the electrode resulting in PtSi/Ir/IrOx (50/200/350 nm) and Pt/Ir/IrOx (25/200/350 nm) stacks for the *BRM* and *research* metallizations, respectively. The Blackrock metallization was very briefly (< 1 min) annealed using a “push–pull” process in a tube furnace operated at 475 °C in an uncontrolled ambient and is designed to aid electrical contact formation and film adhesion. The *research* metallization was annealed in a tube furnace at the same 475 °C but in a pure O_2_ environment for 40 min and is designed to drive the metallurgical reaction at the interface to form platinum silicide (PtSi) for electrical contact formation and adhesion. These processes occur in O_2_-containing-ambients to prevent reduction of the IrOx layer. The character of the metal–semiconductor electrical contacts was not measured, as this is exceptionally difficult with the geometry of the electrode shanks, and no viable method to perform this accurately has been developed.

### Optical and electron microscopy

Optical microscopy was performed with a digital optical microscope (Keyence VHX-5000, Japan) and calibrated measurements were performed using the instrument’s software. Optical microscopy was used to quantify the length of the tip deinsulation of all viable electrodes (*N* = 63) analyzed in this study, and to verify the electrodes were intact and of comparable quality. The geometrical surface area estimated as the surface of a cone with tip deinsulation lengths averaging between 60 and 75 µm is approximately 2 to 4 × 10^–5^ cm^2^, with a surface area of 3.5 × 10^–5^ cm^2^ used for cathodic charge storage capacity (CSCc) and charge injection capacity (CIC) quantification.

Scanning Electron Microscopy (SEM) was performed with an FEI Quanta 600 FEG-ESEM (Thermo Fisher Scientific Hillsboro, OR) instrument using backscattered imaging. Backscattered imaging contrast is sensitive to atomic number (Z), which enables the constituent materials of the UEAs including IrOx, silicon, and Parylene-C to be readily distinguished. BSEM images were acquired at three magnifications from each electrode tip both before and after stimulation, and images at 1250 × are presented for comparison.

### Electrochemical impedance spectroscopy

The EIS measurements presented were collected with a research instrument grade Gamry 600 potentiostat/galvanostat (Gamry, Warminster, PA). Measurements from each electrode were collected before and after the stimulation protocol using a custom 16-channel connector, with the electrodes immersed in phosphate-buffered saline (Gibco PBS, Thermo Fisher Scientific) with physiological osmolality (0.15 mM) and a *p*H of 7.4 using a macro-scale Pt 0.4 mm wire combined counter and Ag|AgCl reference electrode in a 3-electrode-configuration. Measurements were taken from 1 to 10^5^ Hz at 10 frequencies per decade, and the magnitude and phase spectra from before and after stimulation were plotted.

### Cyclic voltammetry and voltage transients

Cyclic voltammetry (CV) measurements were performed using a Gamry 600 potentiostat/galvanostat (Gamry, Warminster, PA) by sweeping the voltage of the electrodes at a constant rate to assess the resultant current flow between the working electrode (WE), counter electrode (CE), and reference electrode (RE) in a 3-electrode-configuration similar to the EIS measurements. The voltage range applied was −0.6 to + 0.8 V vs Ag|AgCl starting at and returning to 0 V to stay inside the theoretical thermal limited water reduction and oxidation reactions. A moderate sweep rate (50 mV/s) was used to evaluate electrochemical reactions and determine the Cathodal Charge Storage Capacity (CSCc) of each electrode. The cathodal region of the CSC is chosen for analysis to enhance the comparison to the stimulation pulses cathodic phase that is primarily responsible for nerve activation. The CSCc was calculated by the following Eq. (Slavcheva et al. 2004) which integrates the cathodal current density and divides by the sweep rate and surface area:

1 $$\text{CSCc = }\frac{1}{{\textit{vA}}}{\int }_{{\text{E}}_{\text{c}}}^{0}\text{|}{\textit{i}}\text{|}{\text{d}}{\textit{E}}\text{ (C }{\text{cm}}^{-2}\text{) }$$

where *E* is the electrode potential (V vs. Ag|AgCl), *i* is the measured current (A), E_*a*_ is the cathodic potential limit (V), *A* is the surface area of the exposed tip (cm^2^), and *v* is the scan rate. The CV and CSCc (mC/cm^2^) are available for three of four devices and precision cathodic CIC (mC/cm^2^) measurements from two of the four devices. Voltage transients (VTs) were longitudinally collected during the stimulation of the electrodes in a 2-electrode configuration (WE and CE), and the E_mc_ and E_acc_ calculated from the voltage waveforms. A Grubbs statistical test was used to account for outliers of CIC measurements. with a one-way ANOVA was used to calculate statistical differences in OriginLab (OriginLab, Northampton, MA).

### Stimulation-stability

**Fig. 1:**
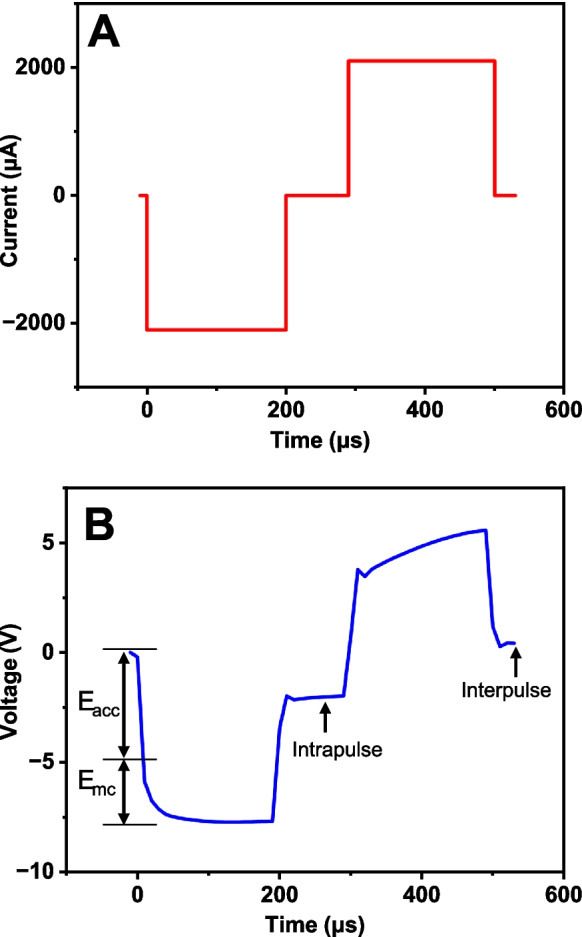
Current pulse waveforms applied to 4 × 4 UEAs and their resultant voltage waveforms through 1 million pulses. **A)** Applied 2,100 *μ*A biphasic current pulse with 200 μs per phase 420 nC/ph, 100 μs intrapulse period, and 2833.3 μs interpulse period. **B)** Representative recorded voltage waveform. Maximum cathodic potential (E_mc_) and access potential (E_acc_) are shown

The Stim-Stab protocol involves stimulation of electrodes with a neural stimulator and collecting voltage transients longitudinally to evaluate the electrode lifetime. This study stimulated a total of *n* = 58 electrodes (6 electrodes from one *research* UEA were not tested) using a research grade stimulator (Multichannel System STG 2008, Germany) where 2 channels were ganged together to enable stimulation at 2,100 *µ*A per phase. Cathodic-leading biphasic pulses with 200 µs per phase delivered 420 nC/ph, with a 100 µs intrapulse duration were applied at the rate of 300 pulses per second (pps or Hz) resulting in a 2,833.3 µs interpulse interval. The resulting pulse morphology is depicted in Fig. 1 below.

The Blackrock standard practice electrodes were pulsed for 1 million pulses, and the research samples were pulsed initially for 1 million pulses, which was extended to 4 million pulses due to the lack of failure observed from the voltage transient data. Voltage transients were recorded with a NIDAQ research grade analog-to-digital converter (NIDAQ USB-6259, Austin, TX) with an input range of ± 10 V and a sampling rate of 100 ksps. Dataset sizes were kept manageable by recording 1 voltage transient out of every 1000 pulses for a total of 1000 and 4000 measurements, respectively, from the *BRM* and *research* arrays. Voltage transients were collected and processed, and the E_mc_ and E_acc_ calculated using custom MATLAB (Mathworks, Inc., Natick, MA) code. The resulting E_acc_ and E_mc_ voltages are plotted longitudinally as a function of pulses. The E_acc_ voltage is the electrode access resistance, or the voltage drop from the spreading impedance of the electrolyte. The E_mc_ voltage is the maximum cathodic voltage at the electrode interface that can show the effects of electrode polarization. The cathodic voltage is commonly chosen over anodic as it is primarily responsible for nerve activation (Cogan 2008). Large, abrupt, and sustained increases in voltage are quantified as the electrical failure point for the electrode, and the resulting lifetime in pulses was recorded. A lifetime plot for the electrical failures was created, and a log-rank statistical analysis was performed using Prism (GraphPad, La Jolla, CA).

## Results

**Fig. 2:**
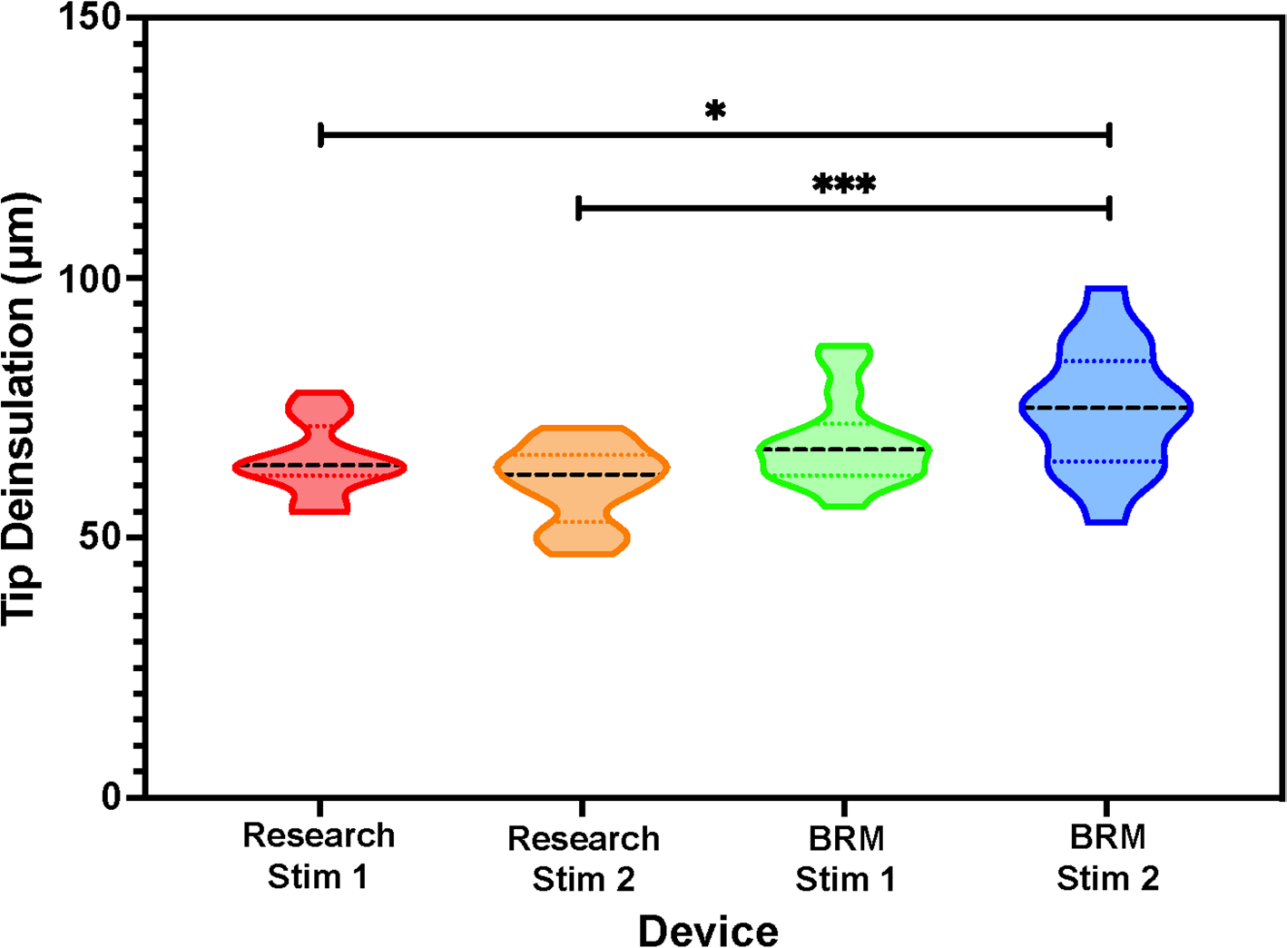
Violin plot showing the tip deinsulation distribution and statistical comparison between the Research and BRM standard-practice 4 × 4 UEAs. Both BRM arrays had larger tip deinsulations, with one statistically significantly larger when tested with a one-way ANOVA

Tip exposure of the Utah Electrode Array (UEA) defines the size and geometrical surface area of the electrode tip and is a critically important factor for the stimulation lifetime of electrodes. Larger tip deinsulations often result in greater stimulation lifetime due to lower voltages and current/charge densities. The tip deinsulation of the two research arrays and two standard practice arrays were carefully measured and statistically compared in the violin plot presented in Fig. 2 The Blackrock standard-practice UEAs had either slightly or statistically significantly larger tip deinsulation, resulting in a comparable or larger electrode surface area (one-way ANOVA). This indicates that the Stim-Stab test has comparable samples, with a potential advantage for the Blackrock devices due to the larger tip area.

**Fig. 3:**
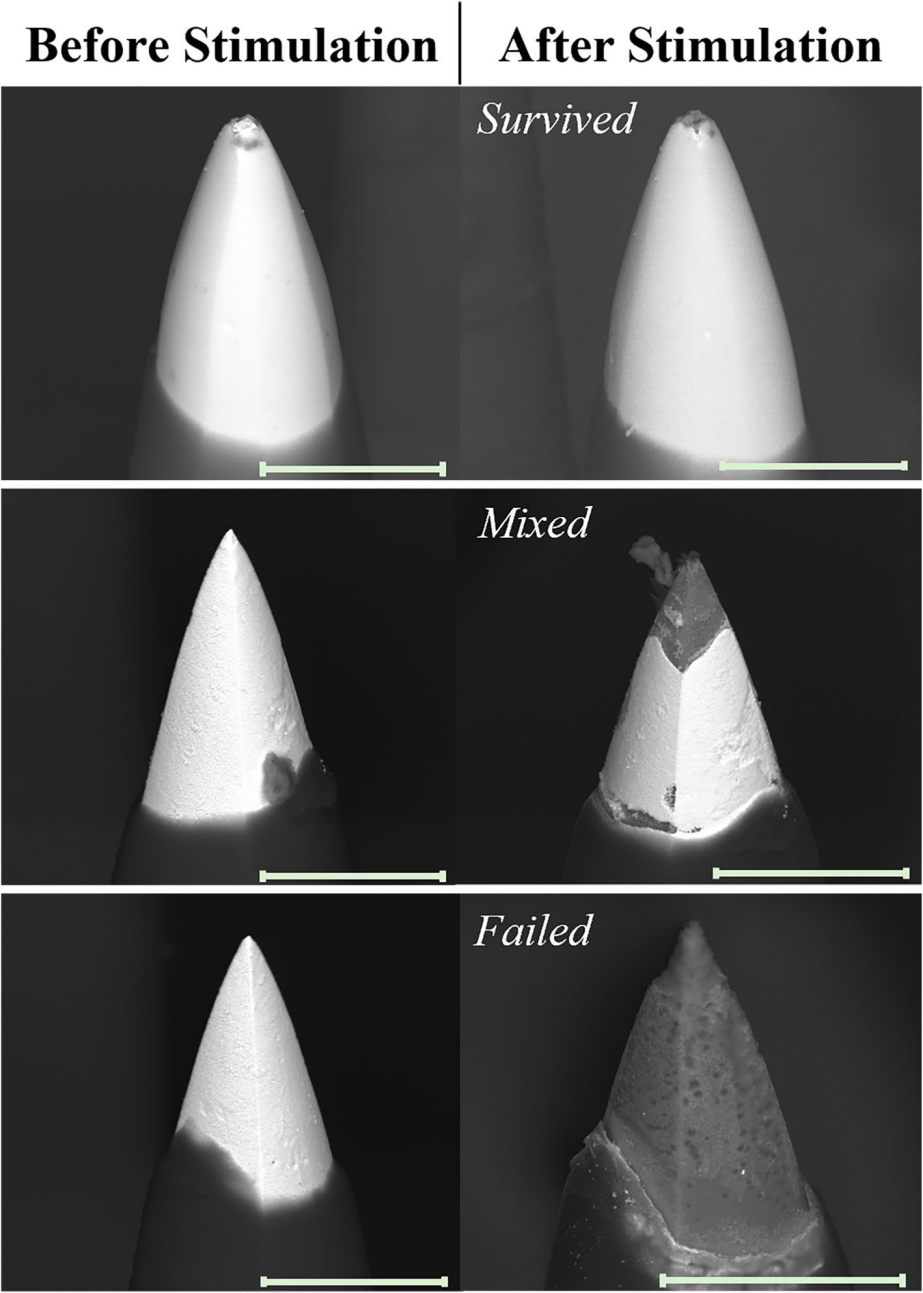
Representative BSEM micrographs of electrodes (left) before and (right) after stimulation. Rows show electrodes with outcomes of (top) *survived*, (middle) *mixed*, (bottom) *failed*. Scale bar = 40 µm. Supplemental Figs. 1 and 2 (S1 and S2) include additional representative BSEM images of devices and electrodes

Backscattered scanning electron microscopy (BSEM) was used to image each electrode both as-fabricated (prior to testing) and after stimulation and representative images are presented in Fig. 3. More comprehensive BSEM characterization of the arrays and electrodes from two devices are presented in supplemental Figs. 1 and 2 (S1 and S2), which highlight the changes in electrodes that occur due to stimulation. Three cases were identified, based on a combination of the BSEM imaging and outcomes from the Stim-Stab protocol, which include *survived*, *mixed*, and *failed*. The *survived* category indicates that minimal metallization degradation is observed (Fig. 3 top row), and also no abrupt increases in voltage transients were observed during Stim-Stab testing. The *mixed* category represents where some degree of metallization damage was observed in BSEM (Fig. 3 middle row), but no electrical failure was noted in the VTs. The *failed* category represents nearly complete loss of electrode metallization (Fig. 3 bottom row) *and* electrical failure from the VTs. For the *research* tip metallization, 50% of electrodes that did not exhibit VT failures did have observed metallization damage making them *mixed*. For the *BRM* arrays 80% of electrodes that did not show failure in VTs had observed degradation in BSEM making them *mixed.* The BSEM imaging was performed after stimulation, and therefore has less utility in evaluating electrode lifetime, as only endpoint data is available. However, in conjunction with other characterization techniques, BSEM it can offer insights to possible mechanisms of the metallization damage (Caldwell et al. 2020; Bjånes et al. 2025).

**Fig. 4:**
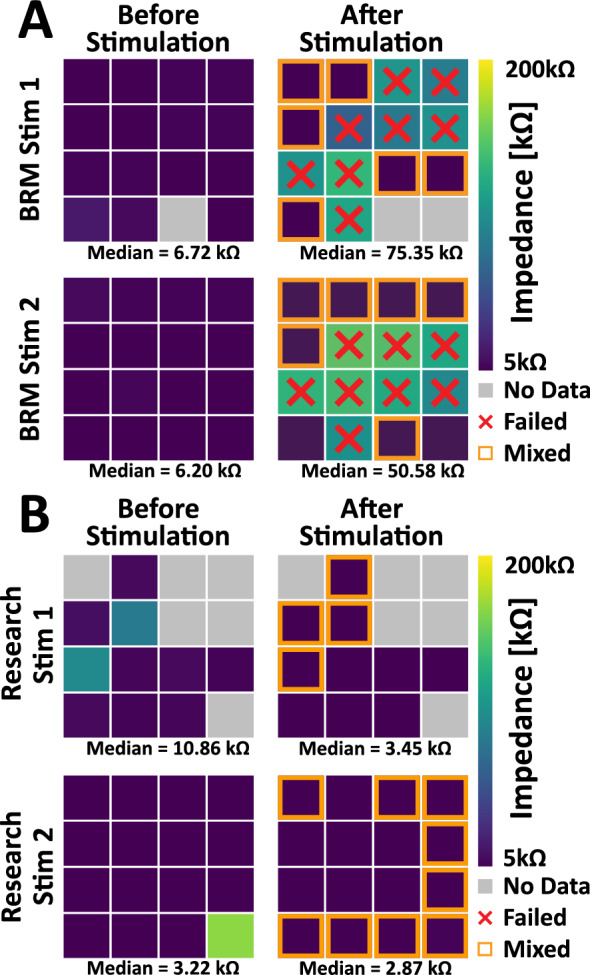
Four 4 × 4 UEAs spatial maps of **A)** *BRM* and **B)** *Research* impedance spectra sampled at 1 kHz. Channel numbering starts from the top left corner, proceeding left to right, top to bottom. Scale bar indicates range of [kΩ] for impedance. Grey boxes designate no data for impedance magnitude (|Z|). A red ‘X’ denotes a failed electrode site meeting the failure criteria of large, abrupt increase in voltage from VT (Fig. 7C). An orange box indicates if the electrode was placed in the “*mixed*” category from longitudinal voltage data and BSEM micrographs

Heatmaps of the 4 UEAs comparing the individual impedance spectra at 1 kHz is displayed in Fig. 4. Each respective electrode from the *BRM* and *research* metallization devices are compared before and after stimulation. The color scale displays impedances ranging from 5 to 200 kΩ, with large impedances associated with failure. It is notable that *Research Stim* devices had three electrode sites with initially high impedance values that lowered after stimulation. This is consistent with literature that *activation* (Otto et al. 2006) can improve electrodes impedance as well as other electrochemical properties. The grey squares in the heatmap represent sites where data was not gathered, while the red ‘X’ represents the electrode sites that were deemed failed from the voltage transient and BSEM imaging data. The after stimulation high impedance values (> 60 kΩ) found on *BRM* devices are concomitant with the *failed* category of electrodes.

**Fig. 5:**
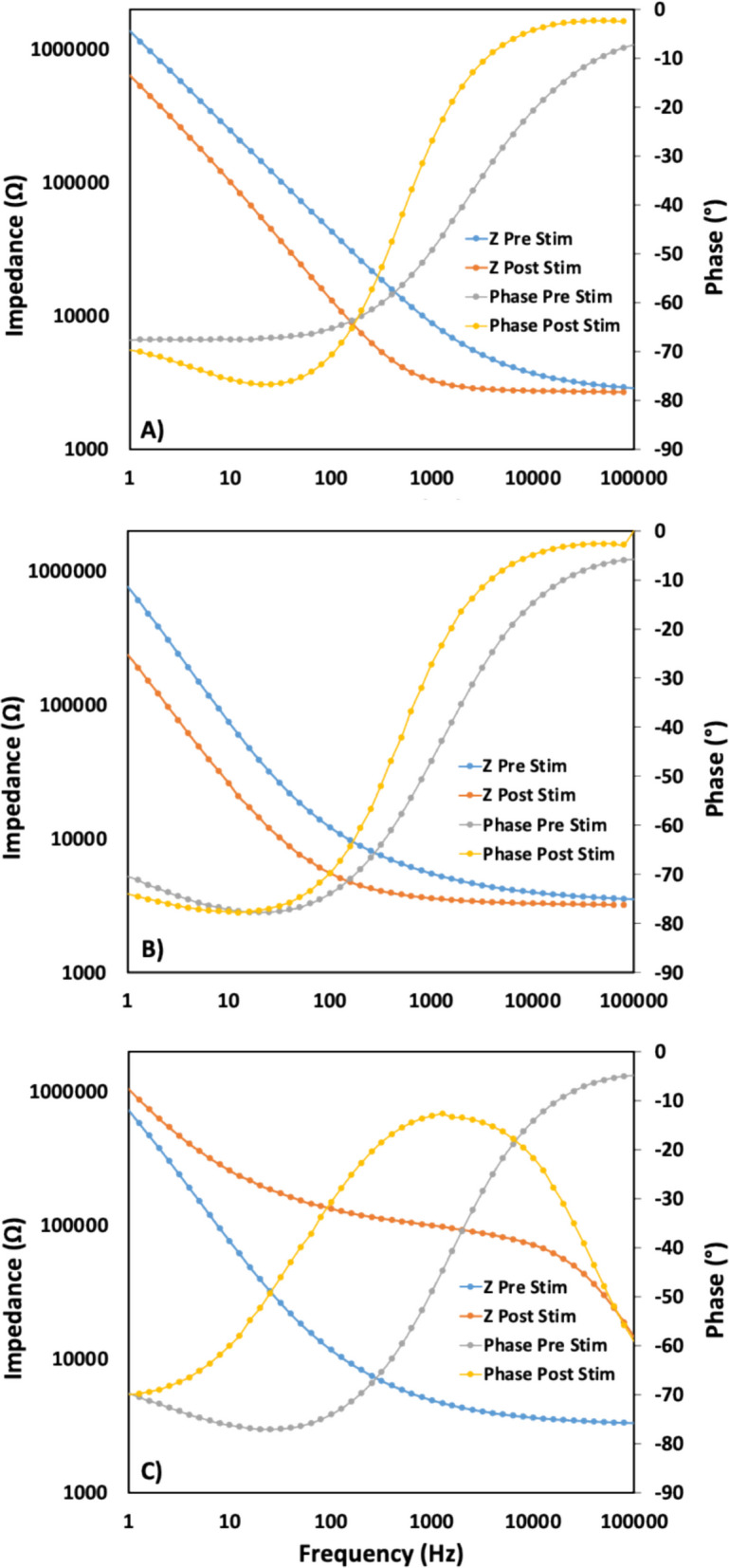
Bode plot of impedance spectra collect from before and after Stim-Stab for the cases of **A**) *su*r*vived*, **B**) *mixed*, and **C**) *failed*. Decreases in impedance are frequently observed for *survived* and *mixed* electrodes, which is attributed to electrode activation. Large increases in impedance are observed for failed electrodes

Although statistical significance was not assessed, spatial information from the *BRM* electrodes could suggest a trend that the innermost devices classified into *failed*, while the *Research* electrodes showed the inverse pattern with the outermost electrodes reaching the *mixed* category compared to the more stable inner electrodes. Outer electrode damage could have a higher chance of being mechanically damaged by outside perturbations during fabrication or stimulation. Electrochemical impedance spectroscopy (EIS) measurements were performed before and after stimulating the electrodes. Representative spectra from the three cases of *survived*, *mixed*, and *failed* are presented in Fig. 5 as Bode plots, and changes in the impedance magnitude and phase were evaluated. Decreases in impedance were often observed for frequencies < 1 kHz for electrodes in the *survived* (Fig. 5A) and *mixed* (Fig. 5B) categories, which is attributed to *activation* of the IrOx on the electrodes. The observed decrease in impedance is notable for the *mixed* category electrodes, as the substantial metallization loss observed in BSEM after stimulation is not associated with increases in impedance for these electrodes. The observed impedance phase angle for these electrodes is between −70 and −80° at lower frequencies and transitions to values near 0° at higher frequencies, consistent with constant phase element and resistive behavior, respectively. Dramatic increases in impedance magnitude are observed across the spectra for electrodes in the *failed* (Fig. 5C) category and its associated complete loss of tip metallization. A more complex phase spectra develops in association with the degraded surface, consistent with a change in materials for the electrochemical interface.

**Fig. 6:**
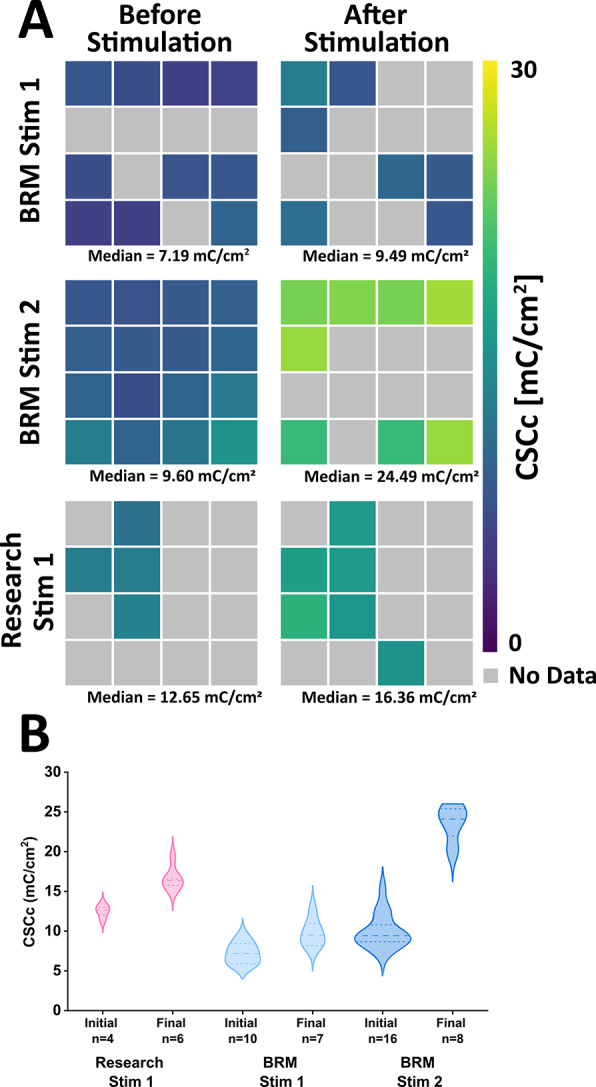
**A)** Cathodic Charge Storage Capacity (CSCc) spatial heatmaps. All median CSCc increased after stimulation, with BRM Stim 2 having a dramatically higher 150% increase post stimulation. Research stim device 2 CSC data was not available. **B)** CSCc violin plots comparing after and before stimulation for each device. All device comparisons have a significant difference between them, with the biggest being BRM Stim 2 after stimulation. Research stim device 2 CSCc data was not available. An average estimated electrode area for the UEAs was 3.5 × 10^–5^ cm^2^

**Fig. 7:**
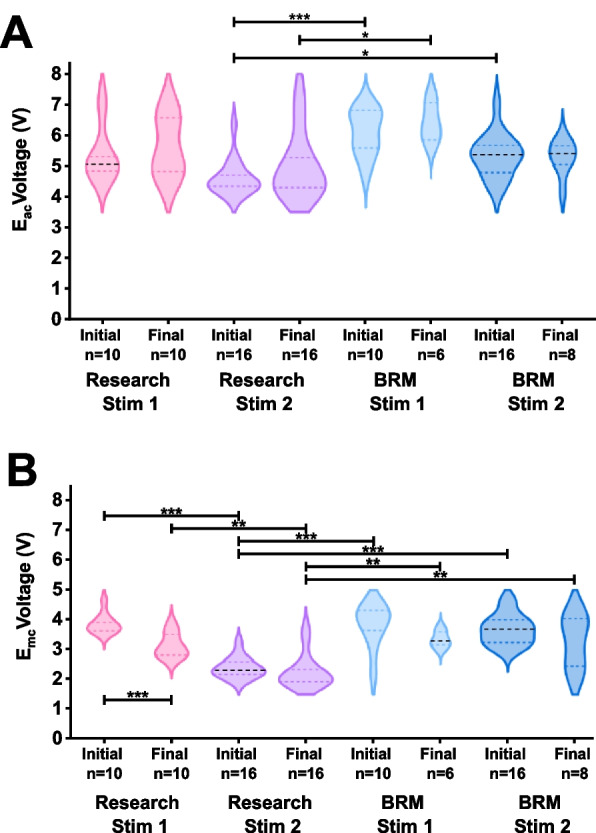
**A)** Violin plot comparing initial vs final measured E_acc_ for each device. **B)** Violin plot comparing initial vs final measured E_acc_ for each device. The Research Stim 2 device was observed to have a statistically significantly lower E_mc_ than other devices. Significance bars on top of the violins signify differences between different devices, while significance bars below the violins compare between the same device before and after stimulation

The charge storage capacity (CSCc) provides information on the type and scale of charge transfer processes for an electrode under quasi-static conditions based on cyclic voltammetry measurements. Representative CV plots from before and after Stim-Stab for *Research* and *BRM* devices are provided in the supplemental figures (Figure S3). No CV data was collected from *failed* electrodes. The CSCc heatmaps for the three devices with available data are presented in Fig. 6A and show that there are no strong variations associated with location. The CSCc values range from relatively modest values of 8 mC/cm^2^ to near 30 mC/cm^2^, with the latter being more similar to but still well below contemporary values for optimized IrOx (Maeng et al. 2020). The heatmaps and violin plots (Fig. 6B) indicate that all measured electrodes increase their CSCc after stimulation, consistent with the decrease in impedances observed for *survived* and *mixed* electrodes and electrochemical *activation*. The *Research Stim 1* device measured had the highest initial CSCc. This device had a higher E_mc_ (Fig. 7B) at the 2100 *µ*A stimulation conditions than the *Research Stim 2* device, suggesting improved charge exchange in the latter device, but CSCc data was not available for comparison. The *BRM Stim 2* device had the largest increase in CSCc with stimulation for those electrode measured after the 1 million pulse endpoint for this device. A concomitant decrease in E_mc_ for this device was not observed from VT measurements presented subsequently, suggesting differences in charge delivery characteristics between quasi-static CV and pulsed VT conditions.

**Fig. 8:**
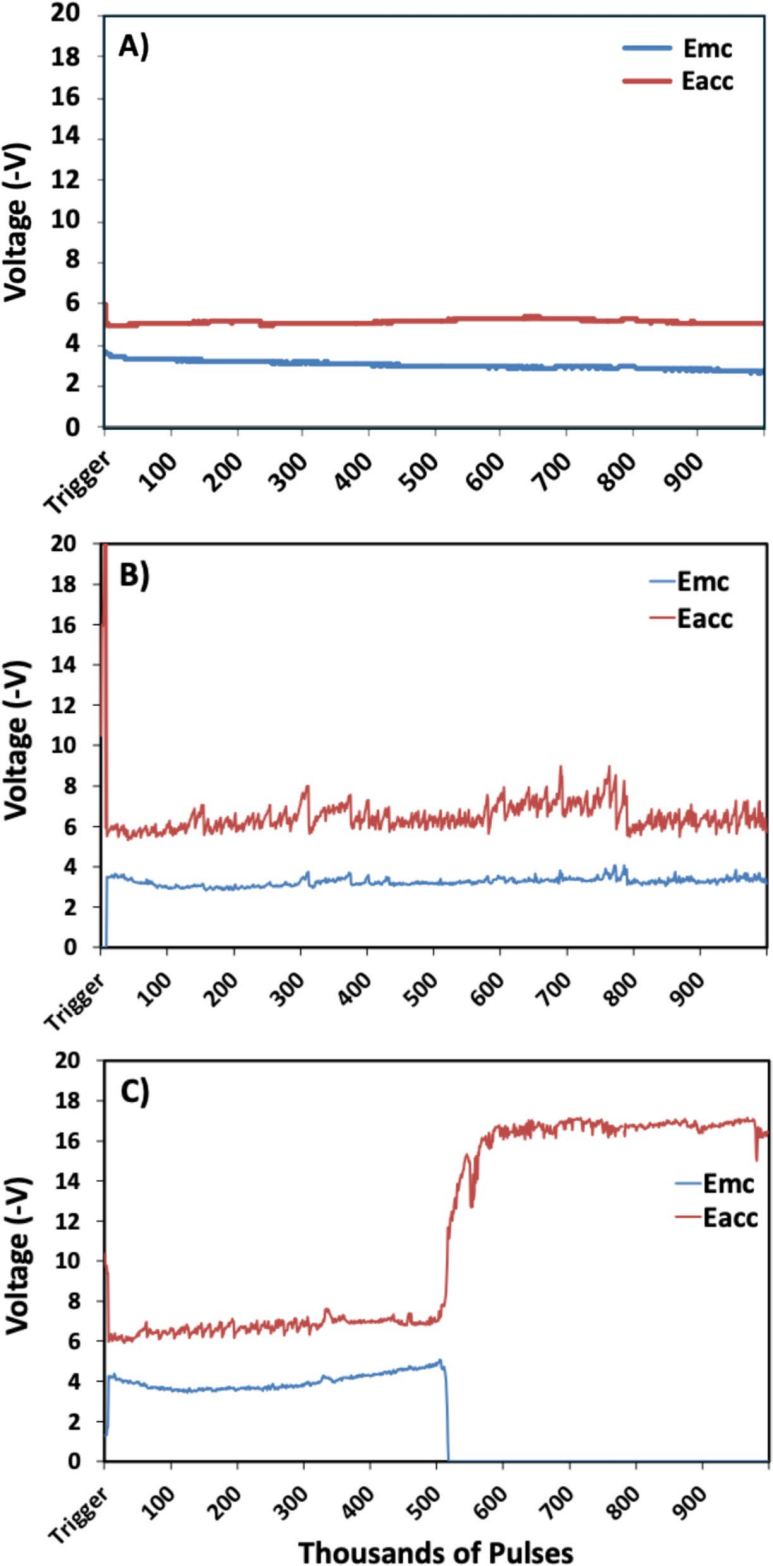
Representative Stim-Stab plots presenting the longitudinal voltage transient characteristics as a function of pulses for samples stimulated for 1 million pulses. Longitudinal voltage transients from samples in the **A**) *survived*, **B**) *mixed*, and **C**) *failed* categories

Representative VT trends are presented from electrodes categorized as *survived*, *mixed*, and *failed* based on the established criteria are presented in Fig. 8. *Survived* and *mixed* outcomes both do not demonstrate abrupt and large increases in voltage transients associated with electrode failure. The failed population shows a large and abrupt increase in the VT and correlates with severe damage to the electrode metallization. The increase in VT can be used to quantify the stimulation lifetime of the electrode. The failed electrode (Fig. 8C) was quantified to have a lifetime of 515,000 pulses as an example for electrode lifetime characterization.

The average charge injection capacity (CIC) for the *BRM* devices were 0.667 ± 0.111 and 1.095 ± 0.235 mC/cm^2^ for devices 1 and 2, respectively, representing a low but typical value. The increased CIC values of *BRM Stim 2* accompanies an increased CSCc (mC/cm^2^) in relation to lower *BRM Stim 2*. The values of *E*_acc_ were largely similar between devices, though there was a statistically significant difference between the mean values for *Research Stim 2* and *BRM Stim 1* devices. This suggests comparable surface areas and spreading impedances in the electrolyte that are responsible for the E_acc_ voltage drop. All devices had a decrease in E_mc_ for *survived* and *mixed* electrodes at the study endpoint of 1 million pulses, consistent with *activation* of the IrOx. This decrease reached statistical significance only for the *Research Stim 1* device. The *Research Stim 2* device had a statistically significantly lower E_mc_ than all other devices, suggesting it possessed the best charge transfer characteristics for this study. However, there is not sufficient data to determine if this is more than a correlation with the optimal Stim-Stab outcomes for this device. It is notable that all E_mc_ values for these high-current stimulation conditions exceeded the water window. These potentials were measured in a two-electrode configuration; therefore, any polarization of the larger counter electrode could contribute to the E_mc_, decreasing the accuracy of this evaluation. However, the E_mc_ in the cathodic 3 to 4 V range are highly likely to have exceeded the water window of −0.6 V. Some electrodes from the *Research* devices tolerated these E_mc_ potentials suggesting resilience of some electrodes outside the water window, conditions often considered damaging to electrodes (Negi et al. 2010a). Large cohorts with more precise measurements are needed to explore this further. Additionally, identifying the underlying mechanisms responsible for the differences in E_mc_ potentials offers the opportunity to further optimize charge transfer processes, and possibly the lifetime of electrodes.

**Fig. 9:**
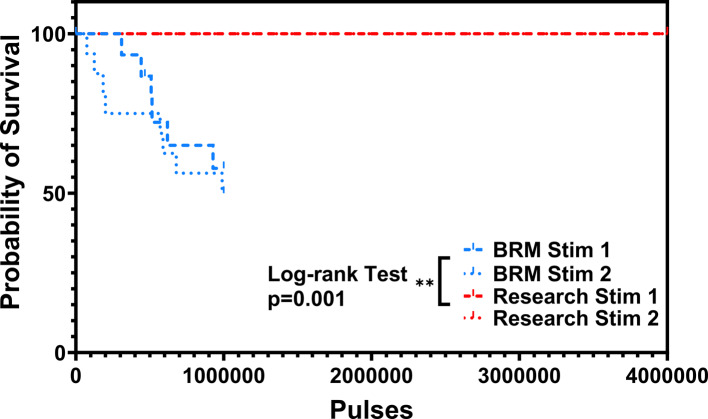
A lifetime plot comparing four 4 × 4 UEAs, two BRM standard practice metallization, and two with *research* metallization. Approximately 50% of BRM metallization electrodes had failed by 10^6^ pulses, whereas no *research* metallization electrodes had electrically failed by 4 × 10^6^ pulses

The number of pulses associated with the point at which the voltage transients markedly increased in magnitude was chosen as the lifetime for *failed* electrodes. These lifetimes were plotted on a per UEA basis so is based on the outcomes from the 16 electrodes per device and are presented in Fig. 9. The Blackrock standard practice electrodes stimulated for 10^6^ pulses and roughly 50% were categorized as *failed*. No electrical failures were noted in the Stim-Stab plots for the *research* metallization arrays at 10^6^ pulses, so additional pulses for a total of 4 × 10^6^ pulses were performed, which also did not result in observed electrical failures. A log-rank test found a highly significant difference between the *BRM* and *research* metallizations, but no significant differences were found within the respective cohorts.

The ability to quantify the failure lifetime for the electrodes provides significantly improved data when compared to pass/fail endpoints more common in stimulation testing paradigms. As previously noted, 80% and 50% of the BRM and *research* metallization electrodes, respectively, which “passed” the Stim-Stab test were observed by BSEM to have notable metallization degradation.

## Discussion

Increasing lifetime of implanted functioning electrodes that are able to record motor system neural signal and evoke sensory percepts through electrical stimulation is paramount for chronic restoration of sensory and motor function. Lifetime evaluation of UEA electrodes with differences in the IrOx metallization was readily quantified using the Stim-Stab protocol for otherwise comparable electrodes. The stimulation acceleration protocol was established as a method to quickly access UEAs metallization capability thresholds on an efficient engineering timescale. Other methods of accelerated aging UEAs have been established in literature such as elevated temperatures or mechanical loadings, but a focused effort is made in this study to only modulate the current amplitude in order to first isolate the effects of stimulation current stress and evaluate stability from the two different fabrication procedures. Abrupt increases in the voltage transient magnitude were found to correspond to drastic delamination of the tip metallization in BSEM imaging and large increases in the impedance magnitude across the spectrum, clear signs of severe electrode degradation. BSEM imaging of some electrodes that did not demonstrate increased voltage transient magnitude highlighted notable degradation had occurred in 50% of the *research* metal samples, indicating the Stim-Stab method provides a more accurate lifetime estimate for severe degradation. Additionally, the impedance magnitude of both *survived* and *mixed* electrode categories decreased during stimulation. This is consistent with electrochemical *activation* of the electrodes, providing additional evidence for this effect, and indicating that changes in electrode impedance must be evaluated in comparison to potential changes in baseline impedance.

These results highlight the large differences in lifetime that can result from changes in the metallization stack. They do not identify the mechanisms for the differences in these outcomes. Pitting observed in BSEM images of silicon exposed by loss of tip metal is consistent with electrochemical degradation and dissolution of silicon. Degradation of silicon was observed in long-term UEA implants of human subjects and was one proposed mechanism for electrode metal loss in that analysis (Bjånes et al. 2025). The strong difference in stimulation lifetimes as a function of the tip metallization reported here suggests that studies to determine the mechanism(s) for degradation would be very useful in the effort to improve the lifetime of UEAs and enhance potential as marketed devices.

The electrode impedances measured before stimulation are notable for mostly being < 10 kΩ, considerably lower than most previously reported impedances for UEAs measured with a precision potentiostat. More similar impedances for an auditory nerve prosthesis application were recently reported, but measured at only 1 kHz with a TDT neural stimulator (Thomas et al. 2024; 2023). Many other recent studies using Neuroport electrodes measured impedances with the Blackrock Cerebus Neural Signal Processor, which does not report or accurately measure impedances < 30 kΩ, often making quality measurements of contemporary UEAs with IrOx difficult. The low initial impedances reported indicate that significant progress to lower electrode impedance has been made. Challenges remain for regularly collecting quality impedance data in vivo, particularly for high channel-count electrode arrays, but this is improving with use of ASIC that include impedance measurements (Konrad et al. 2025; Jung et al. 2024; Jun et al. 2017).

## Conclusion and future work

The stimulation stability (Stim-Stab) method was used to quantify the lifetime and characterize failure modes of a new IrOx tip metallization for Utah arrays compared to the Blackrock standard practice metallization. The *research* metallization was found to have significant improvement in stimulation stability compared to Blackrock current practice metallization in the accelerated aging test. The Stim-Stab method enhances the ability to quantify electrode lifetimes and thereby perform improved statistical comparisons between electrodes and fabrication processes used to make them.

Future work opportunities are revealed from this work center on elucidating the mechanisms for degradation, and how those are impacted by stimulation conditions. Additionally, some data suggests that the nature of the interface between the tip metallization and silicon of the electrode shank is pivotal for decreasing tip metal delamination and improving lifetimes. This work would involve careful characterization and optimization of the electrode surface, metallization deposition conditions, and applied heat treatments, and characterizing the results- a significant work package. More precise three-electrode measurements of potentials during stimulation would allow better mechanistic evaluation of stimulation induced damage. Integration of artificial intelligence to classify the BSEM images would additionally prove useful to precisely pinpoint different failure mechanisms such as delamination, cracking, and electrochemical void formation and allow for less biased quantification.

## Supplementary Information

Supplementary Material 1.

## Data Availability

All data is available upon reasonable request.

## Abbreviations

UEA: Utah electrode array
USEA: Utah slanted electrode arrays
BRM: Blackrock metallization
Stim-Stab: Stimulation stability
BSEM: Backscattered scanning electron microscopy
EIS: Electrochemical impedance spectroscopy
CV: Cyclic voltammetry
CSCc: Cathodal charge storage capacity
WE: Working electrode
CE: Counter electrode
RE: Reference electrode
VT: Voltage transient
E_mc_: Electrode maximum cathodic potential
E_acc_: Electrode access potential
