# Supplementary material for: Improved electrode stimulation stability of Utah arrays*
Source: Bioelectron Med. 2025 Dec 27;11:30. doi: 10.1186/s42234-025-00190-9 (PMC12743391; doi:10.1186/s42234-025-00190-9)
Supplement: Supplementary file 1 — Supplementary Material 1. [file 42234_2025_190_MOESM1_ESM.docx]

**Supplementary Figures**

| 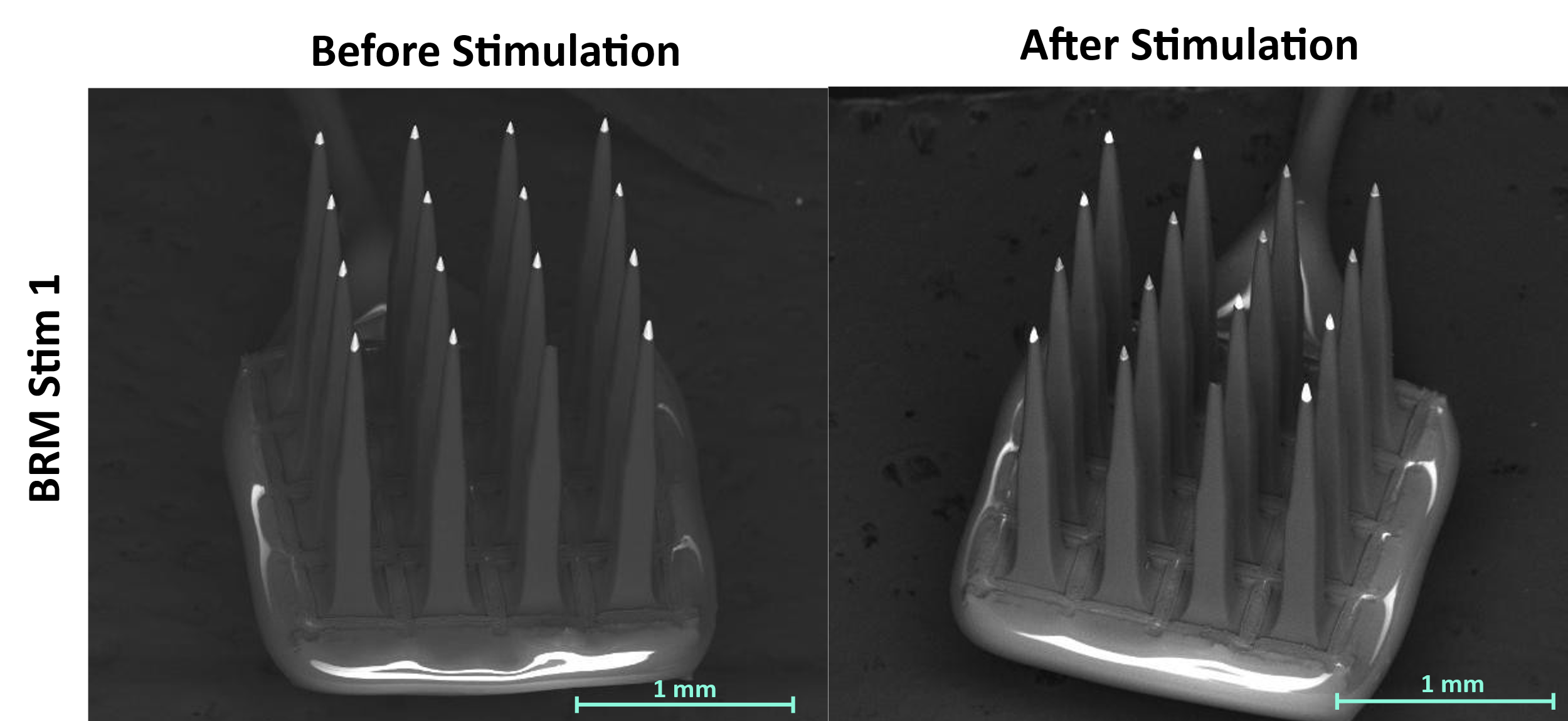 |
| --- |
| 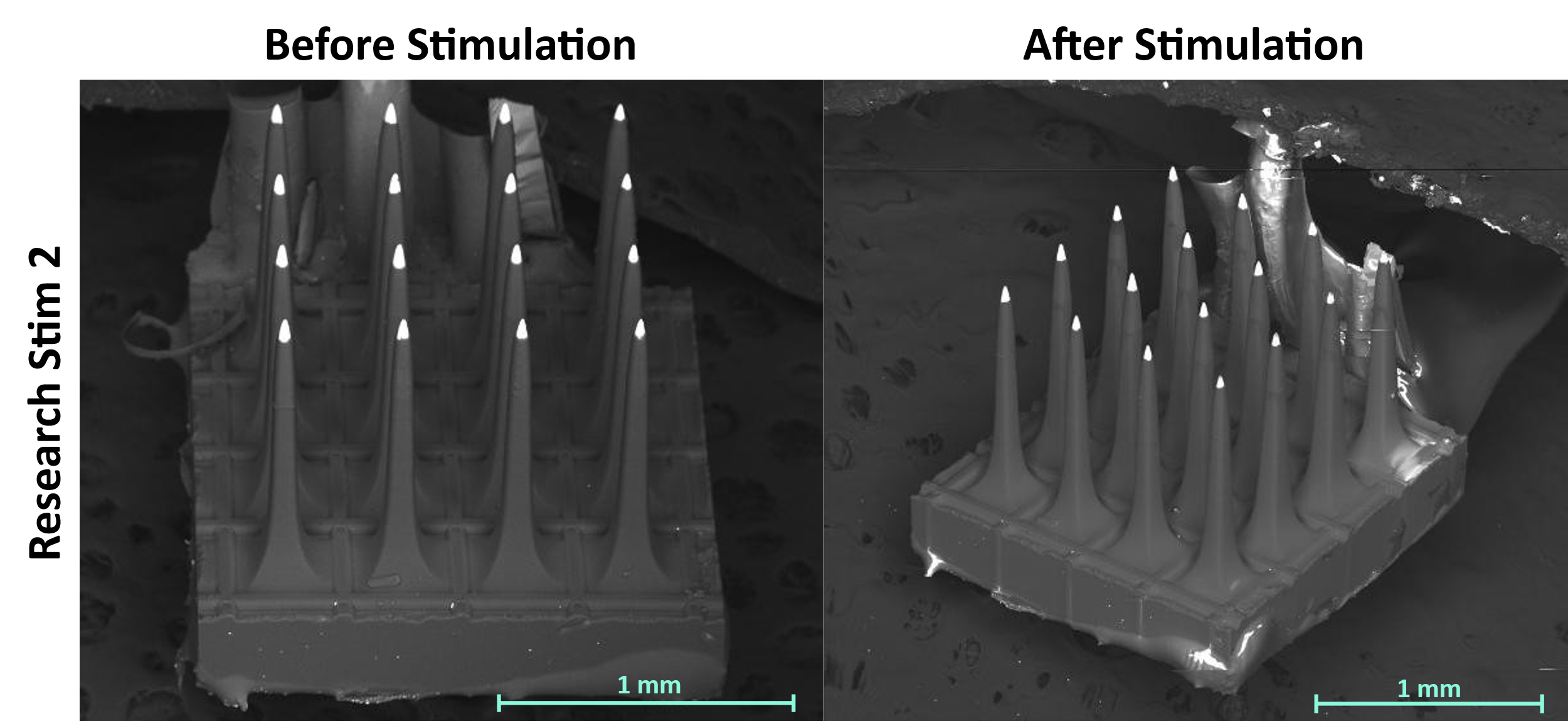 |
| **Figure S1.** Overview BSEM micrographs from two of the four devices. Post stimulation images are in a different orientation but is still representative. Bright regions indicate a higher \|Z\| value, highlighting the IrOx tip capping metallization layer. Darker contrast on the shanks indicates the protective Parylene-C dielectric. Electrode tines are numbered starting from the top left corner, proceeding left to right, top to bottom. |

| **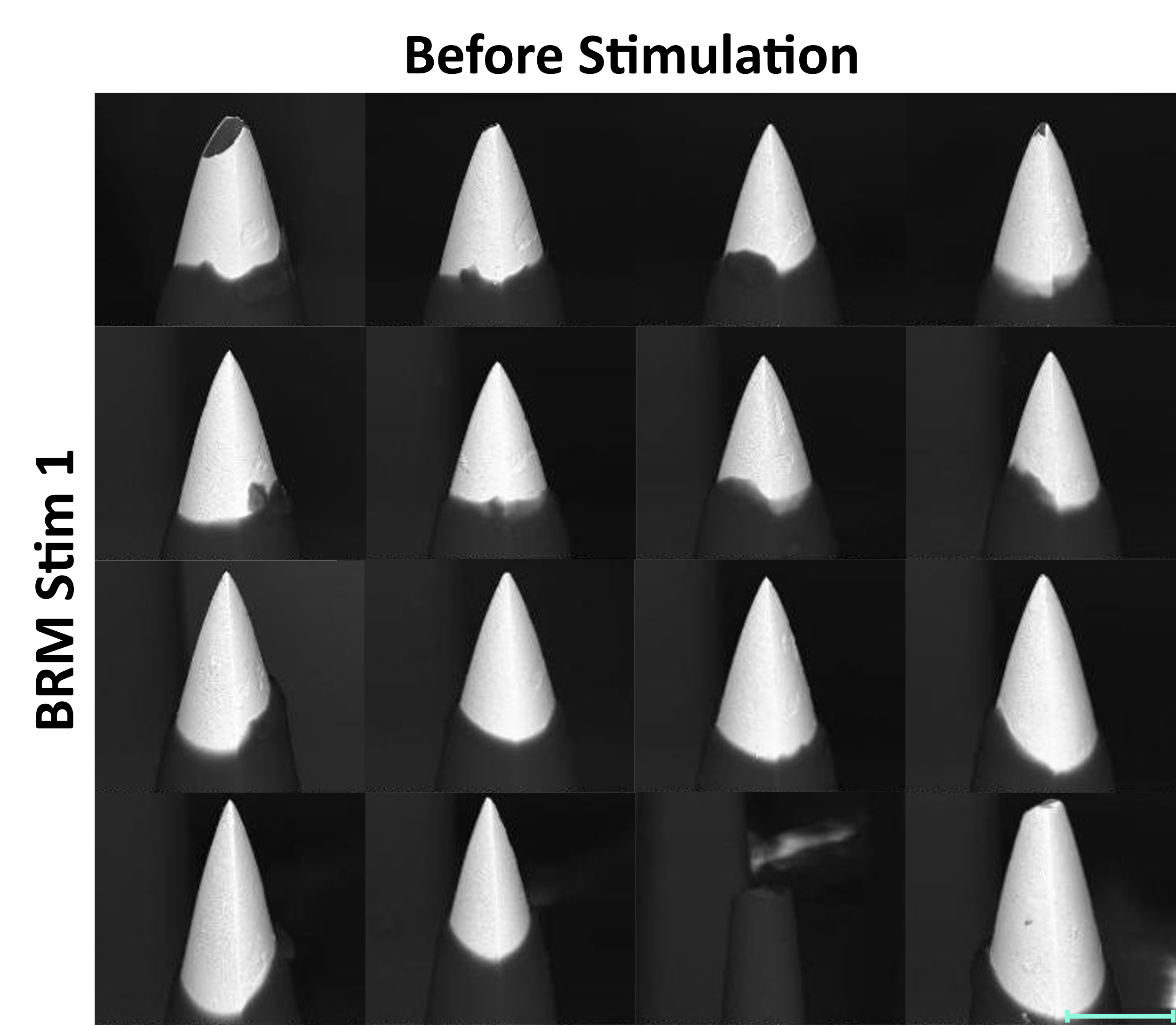** | **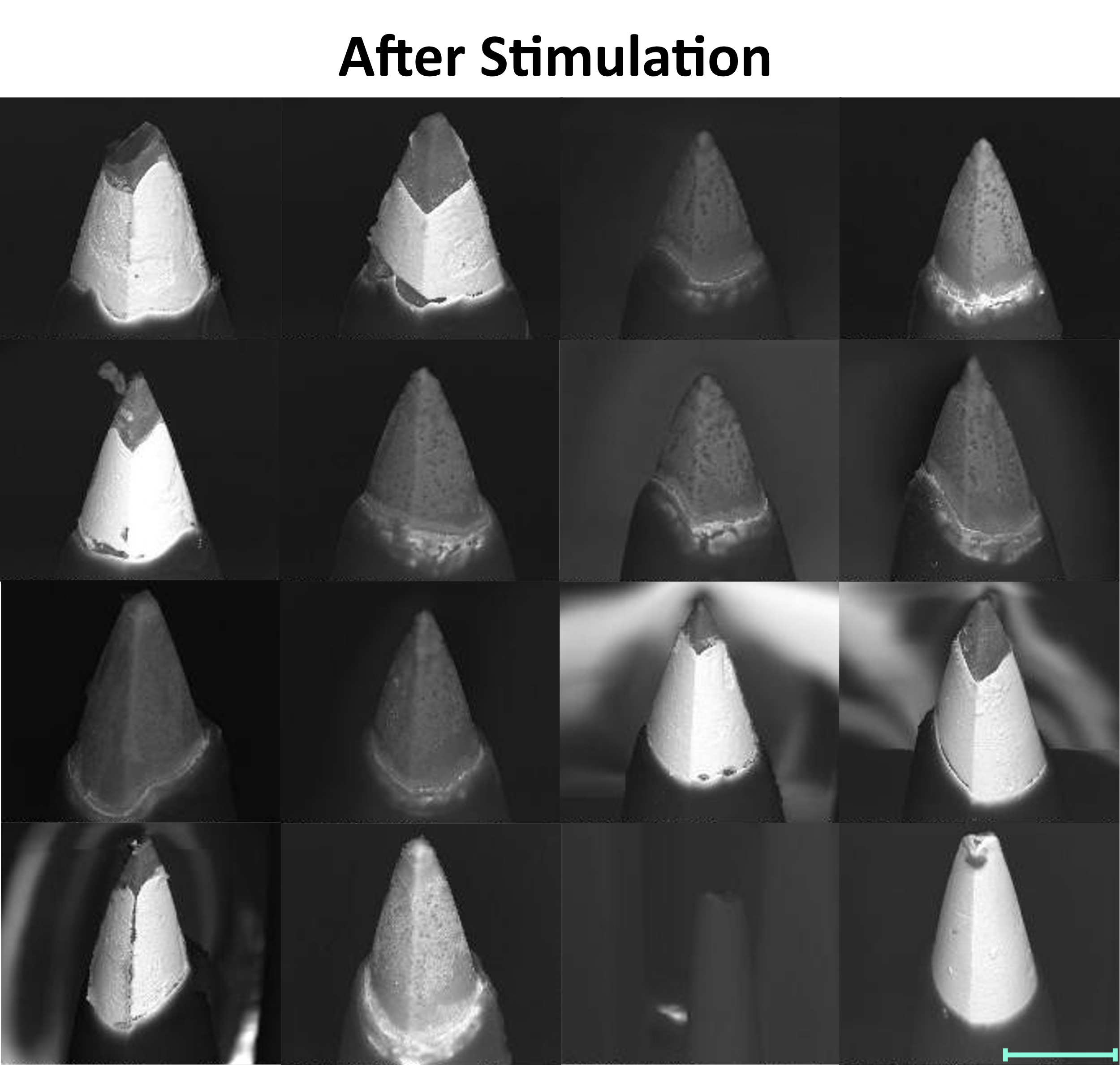** |
| --- | --- |
| **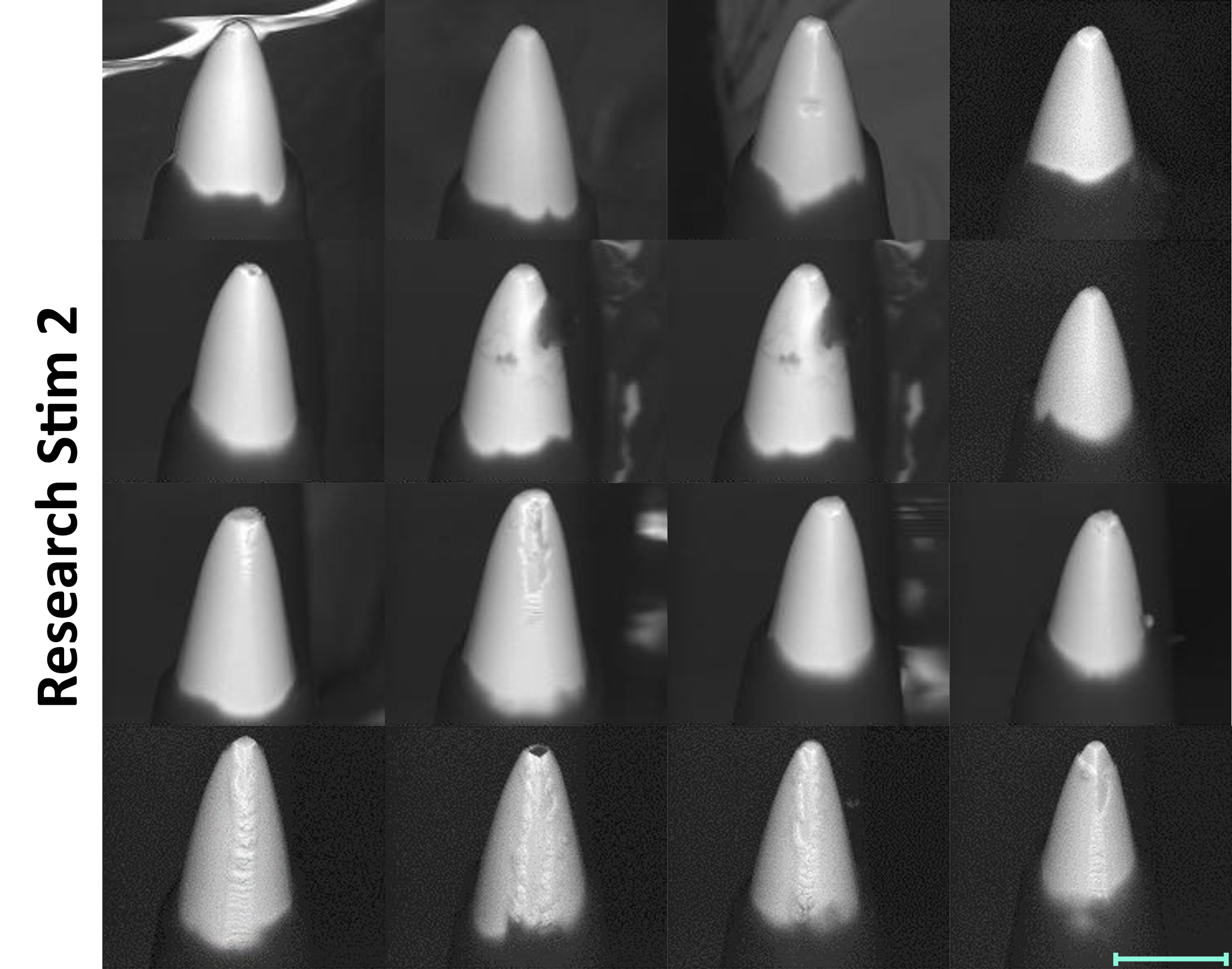** | 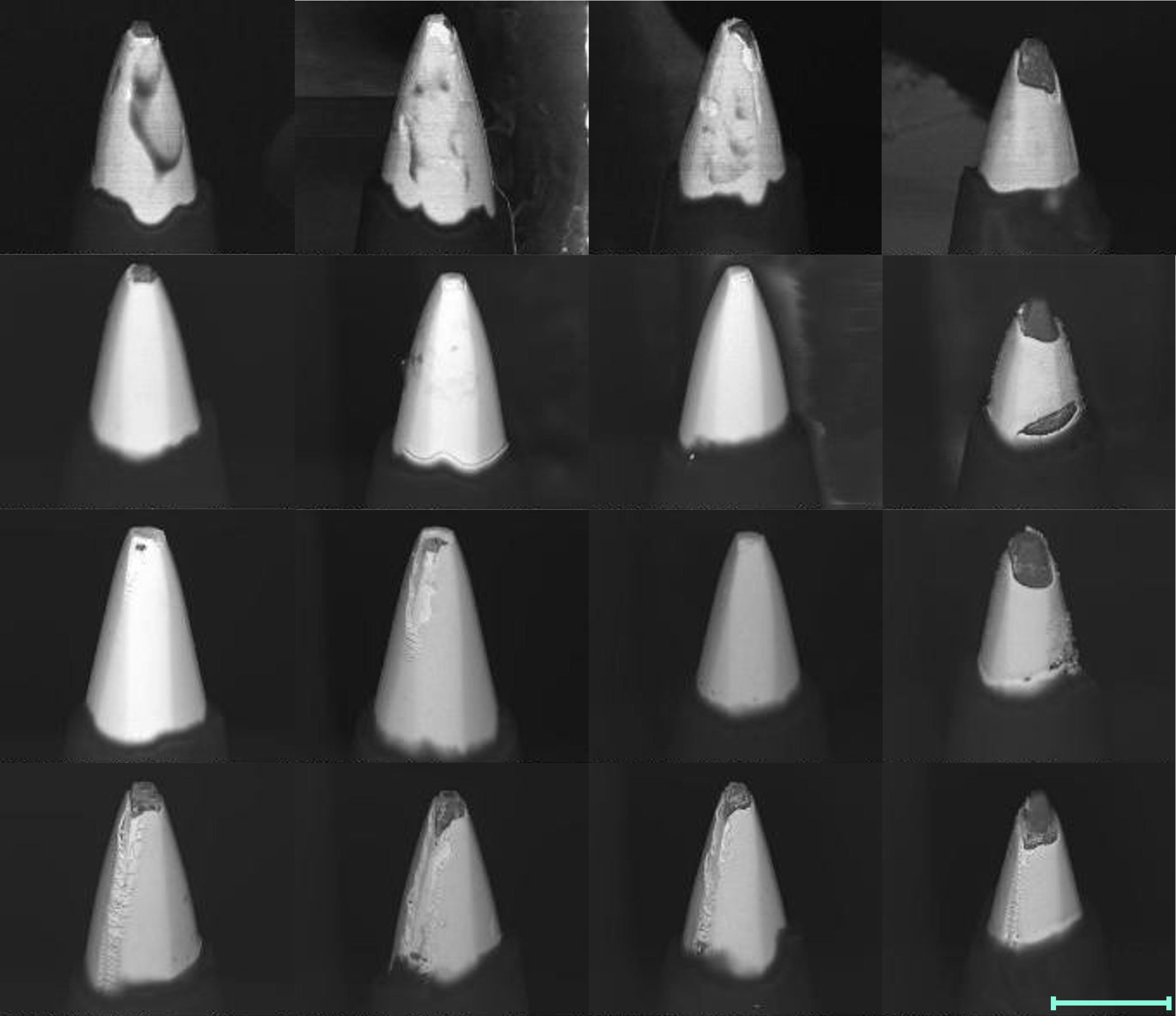 |
| **Figure S2.** BSEM micrographs of individual electrode tines from the overview BSEM images in Fig. S1 above. These micrographs illustrate the full array of the electrode tines stimulation outcomes of survived, mixed and failed. The *BRM Stim 1* device experienced full metal delamination for 53% of the electrodes, 40% electrodes have mixed delamination, and one channel initially mechanically damaged before stimulation. The *Research Stim 2* device had no electrodes with full metal delamination and 56% of electrodes with mixed delamination after stimulation. Scale bar = 40 *μ*m and is representative for all devices. Electrode tines are numbered starting from the top left corner, proceeding left to right, top to bottom. | |

| **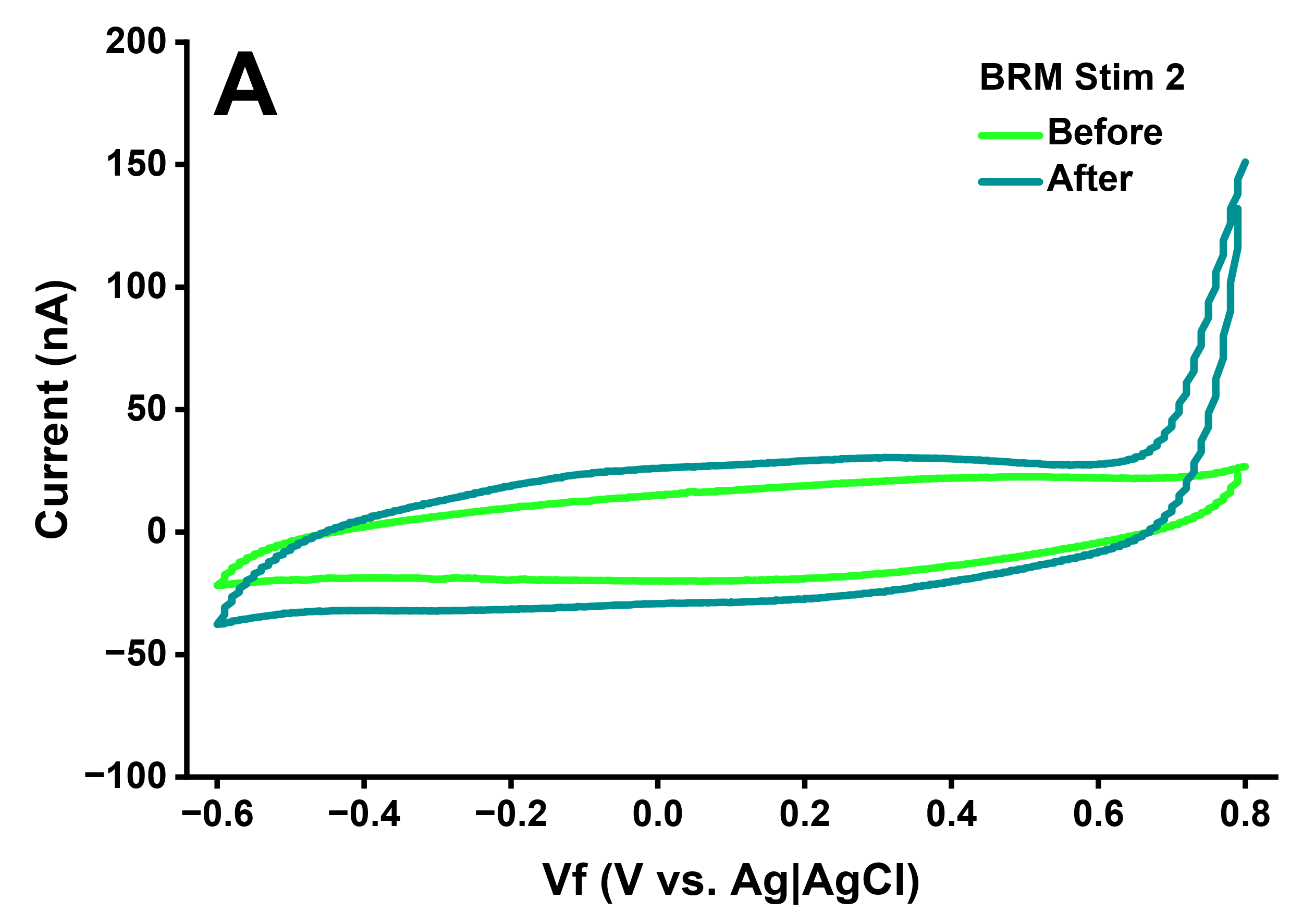** |
| --- |
| **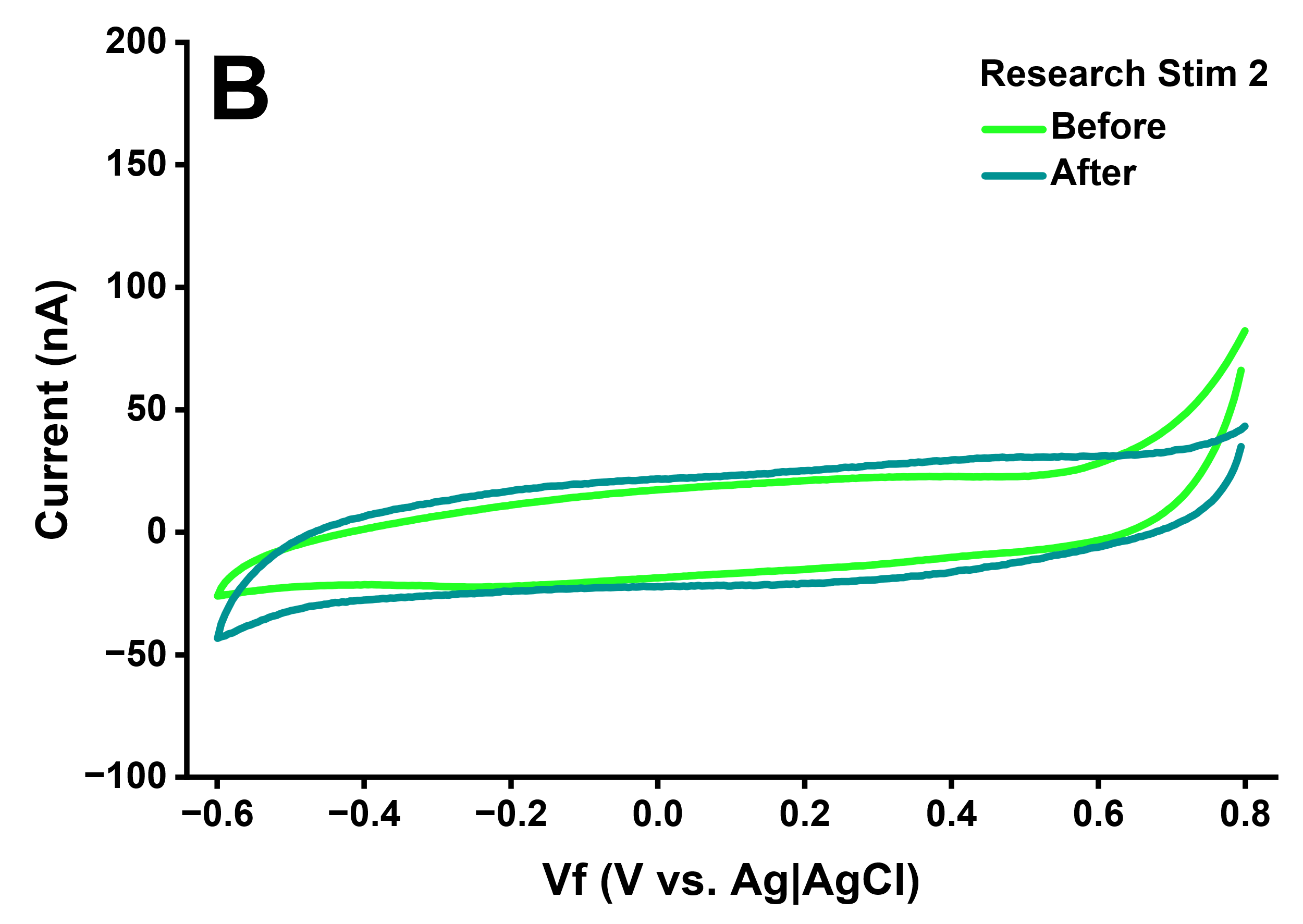** |
| **Figure S3.** Cyclic Voltammograms of a representative *survived* electrodes. A) *BRM Stim 2* electrode before stimulation (CSCc = 12.67 mC/cm^2^) and after stimulation (CSCc = 20.01 mC/cm^2^). B) *Research Stim 2* electrode before stimulation (CSCc = 12.60 mC/cm^2^) and after stimulation (CSCc = 16.57 mC/cm^2^). Both devices after stimulation followed the anticipated tip metal *activation* of increased CSCc reflected in the waveform. |
